# Metagenomic Analysis of the Fecal Archaeome in Suckling Piglets Following Perinatal Tulathromycin Metaphylaxis

**DOI:** 10.3390/ani11061825

**Published:** 2021-06-18

**Authors:** Mohamed Zeineldin, Ameer Megahed, Benjamin Blair, Brian Aldridge, James Lowe

**Affiliations:** 1 Department of Animal Medicine, College of Veterinary Medicine, Benha University, Benha 13511, Egypt; dr.mohamedmeselhy@gmail.com (M.Z.); megahed@illinois.edu (A.M.); 2 Department of Veterinary Clinical Medicine, College of Veterinary Medicine, University of Illinois at Urbana-Champaign, Champaign, IL 61801, USA; bblair2@illinois.edu (B.B.); ba311@illinois.edu (B.A.)

**Keywords:** archaeome, piglets, perinatal, sequencing, tulathromycin

## Abstract

**Simple Summary:**

The archaeal population, or ‘archaeome’, is comprised of unicellular microorganisms with a distinct biology compared with bacteria and has been shown to be an important component of host-associated microbes. While the impact of antimicrobial administration on gastrointestinal microbiota has been widely evaluated, no metagenomics-based analysis has been performed to assess the impact of an early life antimicrobials intervention on the fecal archaeome in swine. The aim of this study was therefore to investigate the impact of perinatal tulathromycin (TUL) administration on the fecal archaeome composition and diversity in suckling piglets using metagenomic sequencing analysis. Our results suggest that perinatal TUL metaphylaxis seems to have a minimal effect on the gut archaeome composition and diversity in sucking piglets.

**Abstract:**

The gastrointestinal microbiome plays an important role in swine health and wellbeing, but the gut archaeome structure and function in swine remain largely unexplored. To date, no metagenomics-based analysis has been done to assess the impact of an early life antimicrobials intervention on the gut archaeome. The aim of this study was to investigate the effects of perinatal tulathromycin (TUL) administration on the fecal archaeome composition and diversity in suckling piglets using metagenomic sequencing analysis. Sixteen litters were administered one of two treatments (TUL; 2.5 mg/kg IM and control (CONT); saline 1cc IM) soon after birth. Deep fecal swabs were collected from all piglets on days 0 (prior to treatment), 5, and 20 post intervention. Each piglet’s fecal archaeome was composed of rich and diverse communities that showed significant changes over time during the suckling period. At the phylum level, 98.24% of the fecal archaeome across all samples belonged to *Euryarchaeota*. At the genus level, the predominant archaeal genera across all samples were *Methanobrevibacter* (43.31%), *Methanosarcina* (10.84%), *Methanococcus* (6.51%), and *Methanocorpusculum* (6.01%). The composition and diversity of the fecal archaeome between the TUL and CONT groups at the same time points were statistically insignificant. Our findings indicate that perinatal TUL metaphylaxis seems to have a minimal effect on the gut archaeome composition and diversity in sucking piglets. This study improves our current understanding of the fecal archaeome structure in sucking piglets and provides a rationale for future studies to decipher its role in and impact on host robustness during this critical phase of production.

## 1. Introduction

The gastrointestinal microbiome has long been known to play major roles in swine health and well-being [1,2,3]. It has been recognized that the piglet’s gastrointestinal ecosystem undergoes a significant shift and dramatic transition from a sterile state to dense colonization during early life [4,5,6]. There is strong evidence that the disturbance of the microbial ecosystem during the suckling period contributes to the disease risk in neonates and can also significantly impact later-life outcomes [7,8]. The swine gastrointestinal tract is a very dynamic and diverse complex ecosystem that hosts several species of microbes, including bacteria, eukaryotes, archaea, and viruses [9,10]. Because resident bacterial populations constitute the most abundant part of the swine microbiome, their impact on swine health and disease has been studied extensively [11,12]. However, there is an urgent need to investigate the interplay between all present members of the microbiota and consequently include community members of other domains of life.

The current advancement of metagenomic methods and optimization of next-generation sequencing protocols have leveraged our current understanding of the dynamics of gastrointestinal archaeal populations and their role in health and disease [13,14,15]. The archaeal population, or ‘archaeome’, is comprised of unicellular microorganisms with a distinct biology compared with bacteria and has been shown to be an important component of host-associated microbes [16]. It has also been shown that archaea are widely distributed, colonize a diverse range of hosts, and reveal body-site-specific patterns [17]. The host-associated archaeome is composed of various lineages that play an important role in biotic methane emissions, removing deleterious microbial metabolites, and inducing host immune responses [18,19]. While the impacts of the archaeome on human health have been questioned for many years [20], its role in swine health and disease remains unclear.

In swine production systems, antimicrobials are still massively used for prophylactic and/or metaphylactic purposes to maintain optimal health and improve growth performance [21,22]. Tulathromycin (TUL) is a bacteriostatic macrolide that acts by inhibiting the biosynthesis of essential bacterial proteins and stimulates the disassociation of ribosomal peptidyl tRNA during the translocation process. On the basis of its favorable antimicrobial characteristics, TUL is utilized therapeutically in neonatal piglets for the control and prevention of infectious diseases at a single dosage of 2.5 mg/kg BW. It has been shown that antimicrobial administration causes deleterious shifts in the structure and functional capacity of the host-associated microbiota [21,22,23]. An early life TUL intervention in piglets exhibited a pronounced shift after a single dose of treatment and had returned rapidly (within two weeks) to a distribution that closely resembled that observed on day 0 prior to treatment [21].

While the impact of tulathromycin administration on the gastrointestinal microbiota has been evaluated [21,22,24], no metagenomics-based analysis has been performed to assess the impact of an early life tulathromycin intervention on the fecal archaeome in swine. The aim of this study was therefore to investigate the impact of perinatal tulathromycin (TUL) administration on the fecal archaeome composition and diversity in suckling piglets using metagenomic sequencing analysis.

## 2. Materials and Methods

### 2.1. Study Populations and Sample Collection

This study was part of a larger study investigating the effect of a single dose of parenteral antimicrobial administration at birth on the developmental dynamics of gastrointestinal microbial ecosystems in piglets during suckling periods. The study procedure and the use of animals were performed according to the recommendations and the guidelines of the University of Illinois at Urbana-Champaign Institutional Animal Care and Use Committee (IACUC Protocol No. 14288). Briefly, a total of 16 sows with their newborn piglets were used in this study. Five days before farrowing, the pregnant sows were transferred to farrowing pens and kept there until the end of the experiment. Sows were given water ad libitum and fed a standard lactation diet via an automatic dry feeding system. No antimicrobials were administered to the enrolled sows before or after farrowing. The teeth of the piglets were not routinely polished, and their tails were not docked. Directly after birth, litters were randomly separated into two different pens and assigned to one of two groups: control (CONT; *n* = 8 L) and TUL (*n* = 8 L). In the TUL group, a total of 108 piglets were treated with 2.5 mg of TUL/kg IM (Draxxin^®^, Zoetis US, Chicago, IL, USA). In the CONT group, a total of 113 piglets were treated with saline (1cc/IM). The TUL-treated piglets were housed together with their mother in a conventional farrowing pen that was approximately 1.9 m by 2.6 m, where the sow was confined so that she could not turn around. Additionally, the sidewall of the pen was solid in order to prevent contact with the other piglets that were assigned to the control group.

A daily physical examination was performed by farm personnel individually to evaluate the health condition of all piglets and their dams. Piglets were individually identified within the litter and weighted on day 0 and day 20. All piglets were weaned on day 21 along with all other litters in the farm that were not enrolled in the study. Individual deep fecal swabs (Pur-Wraps^®^, Puritan Medical Products, Gulford, ME, USA) were collected from two piglets from each litter immediately prior to treatment (day 0) and again on days 5 and 20 after treatment. The fecal swabs were transported in dry ice-chilled boxes to the laboratory on the same day and stored at −80 °C until further processing.

### 2.2. DNA Extraction, Whole Genome Sequencing, and Bioinformatics Analysis

Genomic DNA was extracted from subgroups of piglets across the different time points (CONT; *n* = 4 piglets and TUL; *n* = 4 piglets) and from negative control samples (sterile cotton swabs and extraction kit reagent) using a Power Fecal DNA Isolation kit (MO BIO Laboratories, Inc., Carlsbad, CA, USA) according to the manufacturer’s standard protocol. The fecal swabs were randomly selected from piglets that remained healthy throughout the suckling period. For each sample, the total DNA concentration and integrity were evaluated by optical density using a Nanodrop™ spectrophotometer (NanoDrop Technologies, Rockland, DE, USA) at wavelengths of 260 and 280 nm and agarose gel electrophoresis (Bio-Rad Laboratories, Inc, Hercules, CA, USA). Extracted DNA was immediately stored at −20 °C pending sequencing. All DNA was shipped on dry ice for sequencing at the W. M. Keck Center for Comparative and Functional Genomics (University of Illinois at Urbana-Champaign, Champaign, IL, USA). DNA libraries were constructed using the Nextera DNA Flex Library Preparation Kit (Illumina, Inc., San Diego, CA, USA), and sequenced from both ends following the manufacturer’s guidelines (Illumina, Inc., San Diego, CA, USA).

Raw sequence data files obtained from MiSeq Illumina sequencing were de-multiplexed and converted to fastq files using Casava v.1.8.2 (Illumina, Inc., San Diego, CA, USA). The quality of the sequence reads was assessed using FastQC software [25]. Adaptor sequence and low-quality reads were trimmed from the raw sequence data using Trimmomatic software [26]. Fastq sequence files were then uploaded to the Metagenome Rapid Annotation Using Subsystems Technology (MG-RAST) webserver to determine the taxonomic composition of fecal archaeal communities at both the phylum level and the genus level [27]. MG-RAST utilizes a high-performance data-mining algorithm along with curated genome databases that rapidly disambiguates millions of short reads of a metagenomics sequence into discrete microorganisms engendering the identified sequences. In MG-RAST, sequence reads were subjected to quality control, including removal of sequencing artifacts, removal of host-specific species sequences, length filtering (removal of sequences with a length > 2 standard deviations from the mean), and ambiguous base filtering (removal of sequences with >5 ambiguous base pairs). Normalization was performed using a log_2_-based transformation (log_2_ (x + 1)), followed by standardization within each sample and linear scaling across all samples [27]. Due to the uneven sequencing depth, all samples were randomly subsampled to the sequences per sample that showed the lowest sequence length (Figure S1). We used a non-redundant multisource protein annotation database (M5NR) as the annotation source for microbial classification. Archaeal abundance was analyzed using a best-hit classification approach with a maximum e value of 1 × 10^−5^, a minimum identity cutoff of 60%, and a minimum alignment length cutoff of 15. The fecal archaeome alpha diversity was calculated in PAST version 3.13 using Chao 1 and Shannon indices. The beta diversity of the fecal archaeome was computed using principal component analysis (PCA) based on non-phylogenetic Bray–Curtis distance metrics [28]. To be publicly available, we deposited the whole-genome sequencing data in MG-RAST under the following accession numbers: mgm4779141.3 to mgm4779164.3.

### 2.3. Statistical Analysis

Statistical analysis and graphing were performed using PAST version 3.13 and JMP^®^ Pro 13 (SAS Institute Inc., Cary, NC, USA). The difference in overall archaeal composition between the CONT and TUL groups was determined using non-parametric multivariate analysis of variance (PERMANOVA) with 9999 permutations and Bonferroni-corrected *p* values in PAST version 3.13. The difference in the fecal archaeome relative abundance and alpha diversity metrics between the two groups (CONT and TUL) at each time point (day 0, 5, and 20) were analyzed using a Mann–Whitney pairwise comparison test with sequential Bonferroni significance in PAST version 3.13. The overall fecal archaeome composition between the two groups was assessed using the linear discriminant analysis (LDA) effect size (LEfSe) pipeline using Galaxy (https://huttenhower.sph.harvard.edu/galaxy/, accessed on 1 January 2021). The LEFSe algorithm first used the non-parametric factorial Kruskal–Wallis test to detect taxa with a significantly different abundance, followed by a pairwise Wilcoxon test to detect the biological consistency between the two groups, and then used LDA to estimate the effect size of each differentially abundant feature. All statistically significant differences are stated at *p* < 0.05.

## 3. Results and Discussion

### 3.1. Whole-Genome Sequencing Summary

The development of high-throughput sequencing techniques has greatly improved our understanding of the composition and function of the host-associated microbiome [29]. However, our knowledge of the archaeome structure and how it interacts/communicates with other host microbial members remains questionable. Antibiotic interventions are considered to be one of the most common threats to the microbiome’s ecology as they can substantially disrupt the composition and functionality of the gut microbial community [21]. Although several studies have investigated the impact of antimicrobial administration on the composition and diversity of the swine gut microbiota [21,22,23,30], no information is available on its impacts on the fecal archaeome in neonatal piglets. In this study, we evaluated the effect of an early life TUL treatment on the fecal archaeome composition and diversity in piglets during the suckling period. To the best of our knowledge, this is the first study to use shotgun metagenomics sequencing to study the impact of an early life antimicrobial intervention on the fecal archaeome development in neonatal piglets. Using the criterion of the MG-RAST taxonomic classification, a total of 112,457 sequence reads were taxonomically assigned to an archaeal population (mean number of sequences per sample: 4685.7; median: 4196; range: 16,248–1212), all of which were assigned according to the RefSeq classification.

### 3.2. Effect of TUL Treatment on the Fecal Archaeome Apha and Beta Diversity

**Figure 1:**
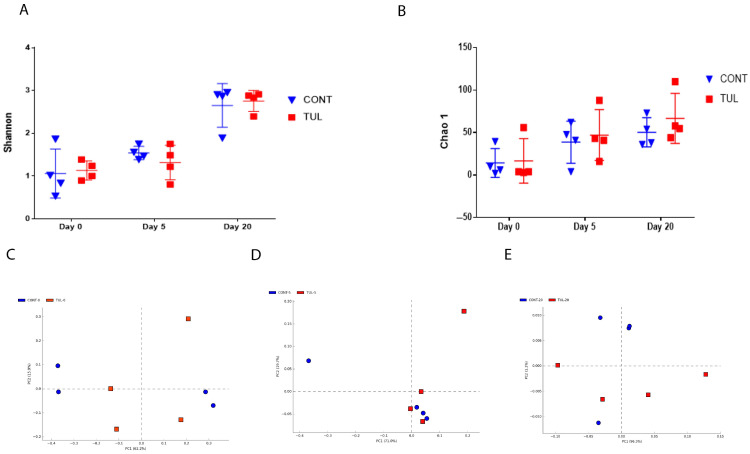
(**A**) Shannon diversity of the control (CONT) and tulathromycin (TUL) gut archeome at 0, 5, and 20 days of life. (**B**) The difference in the Chao 1 index measured between the CONT and TUL groups at different sampling days (0, 5, and 20). Principal component analysis (PCA) based on the non-phylogenetic Bray–Curtis distance metrics for CONT and TUL at days 0 (**C**), 5 (**D**), and 20 (**E**).

We investigated the effects of early life TUL metaphylaxis on the fecal archaeome diversity. The archaeome alpha diversity was computed using Shannon diversity and Chao1 richness metrics. Analyses of archaeal alpha diversity metrics revealed no significant differences between the CONT and TUL groups (Figure 1A,B). In contrast, Shannon diversity and Chao1 metrics showed a significant increase on day 20 compared with day 0 and day 5 (*p* = 0.01 and 0.04, respectively). Similarly, the few studies performed on archaea suggest that the archaeal population displays an overall higher diversity in the older population [31]. To evaluate the potential effect of TUL antibiotic treatments on the overall archaeal population during the first 20 days of life, we compared the microbial community structure (beta diversity) between the two groups at the different time points using the Bray–Curtis dissimilarity metric. Beta diversity analysis (an estimate of the archaeal population’s expression of diversity between different group) revealed that the archaeal community composition was not clustered on days 0, 5, and 20 as shown by PCA of the Bray–Curtis distance (PERMANOVA, *p* > 0.05; Figure 1C–E).

### 3.3. Effect of TUL on Fecal Archaeome Composition

**Figure 2:**
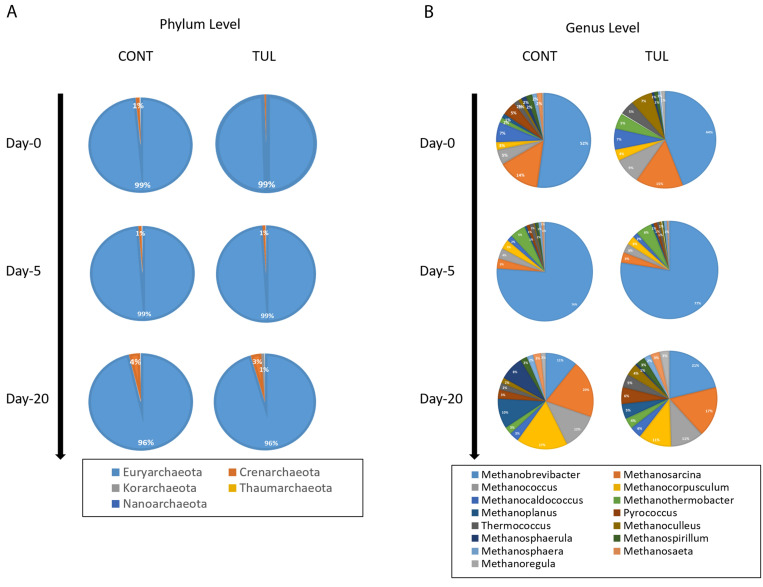
Fecal archaeome taxonomic classification of shotgun metagenomic sequences at the phylum (**A**) and genus (**B**) level for the control (CONT) and tulathromycin (TUL)-treated piglets at days 0, 5, and 20 of life. Only those bacterial phyla and genera that averaged more than 1% of the relative abundance across all samples are displayed.

In terms of relative abundance, a total of 5 archaeal phyla and 60 archaeal genera were detected using the MG-RAST webserver. Similarly to previous metagenomics-based studies of the gastrointestinal archaeal population [16,17,32], the most abundant archaeal phylum across all samples was *Euryarchaeota*, representing 99% and 96% of the sequence reads in the CONT and TUL groups at days 0 and 20, respectively (Figure 2A). Several other archaeal phyla (*Crenarchaeota, Korarchaeota, Thaumarchaeota,* and *Nanoarchaeota*) were identified in a lower frequency and abundance in both the CONT and TUL groups. Our results show that there was no significant change in the fecal archaeome composition at the phylum level between CONT and TUL-treated piglets. However, TUL-treated piglets exhibited a numerical increase in the very rare archaeal phylum *Korarchaeota* at day 20 post intervention (0.16% vs. 0.97% in CONT piglets, *p* = 0.12, Figure 2A).

At the genus level, the predominant archaeal genera at the baseline (day 0) were comprised of common archaeal genera, including *Methanobrevibacter* (44.64%), *Methanosarcina* (13.68%), *Methanocaldococcus* (6.41%), *Methanococcus* (6.36%), *Methanoculleus* (3.78%), *Methanothermobacter* (3.18%), *Thermococcus* (3.01%), *Pyrococcus* (2.33%), *Ferroplasma* (2.07%), *Methanospirillum* (1.57%), *Methanosphaerula* (1.14%), *Methanosphaera* (1.09%), and *Methanocorpusculum* (1.01%). Similarly, *Methanobrevibacter* species are the predominant archaea in the gastrointestinal tract of various species, including ruminant and non-ruminant species [31,33]. The presence of the highly abundant methanogens has been also confirmed in the human gut, with *Methanobrevibacter* being the most abundant methanogenic archaeon found in stool specimens [15,34]. Other methanogenic archaea, including *Methanosphaera*, *Methanomassiliicoccus, Methanimicrococcus,* and *Methanosarcina* species, have also been identified in various animal species, but they are usually less abundant [31,33]. Methanogenic archaea are considered to be almost ubiquitous inhabitants of the gastrointestinal microbiome and are functionally important due to their role in molecular hydrogen consumption, energy metabolism, and adipose tissue deposition [33]. In contrast to the beneficial archaeal population, the ability of certain archaeal species to produce methane may play a role in the pathogenesis of several gastrointestinal diseases [35]. Besides methanogenic archaea, haloarchaea and thaumarchaeota have also been reported in our study. Thaumarchaeota (also referred to as *Nitrososphaeria*) and halophilic archaea (particularly *Halococcus*) are mainly associated with the host’s outer or exposed surfaces and have been shown to exhibit specific functions that can either protect or negatively affect the host [36]. The function of halophilic and thaumarchaeota archaea in piglets remains elusive and their presence in the piglets’ fecal samples raises many questions about the origin of these archaea.

**Figure 3:**
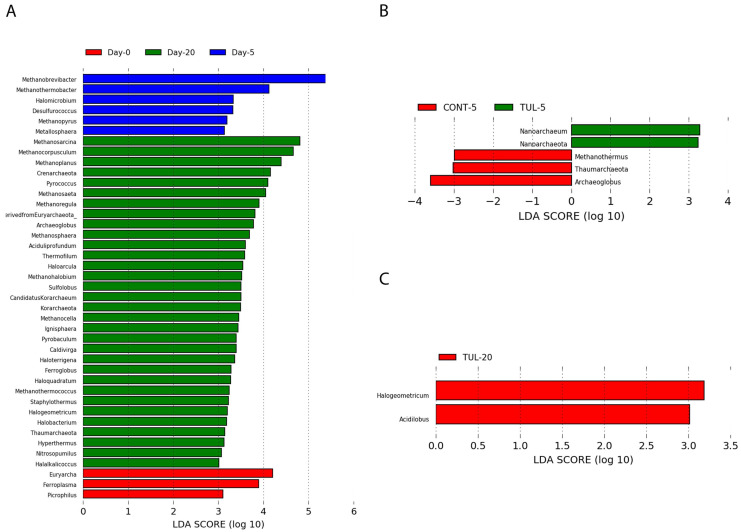
(**A**) Identification of indicator archaeal taxa associated with a statistically significant differential abundance between the different sampling days (0, 5, and 20). Identification of indicator archaeal taxa associated with a statistically significant differential abundance between the control (CONT) and tulathromycin (TUL)-treated piglets at days 5 (**B**) and 20 (**C**) of life. The top OTUs with the highest LDA score log_10_ > 2.0 that discriminate between the CONT and TUL-treated piglets at each time point are depicted.

Despite the functional potential of the archaeome, the characteristics of archaeal neonatal colonization and its developmental dynamics remain limited. The distribution of the most abundant archaeal genera in both the CONT and TUL groups on different sampling days are depicted in (Figure 1B). Collectively, the archaeal sequence data analysis revealed that the archaeal composition at the genus level in CONT and TUL varied greatly according to age (Figure 2B). The development of fecal archaeal communities during the first 20 days of life across all samples was also evaluated using the LEfSe algorithm (LDA cutoff score of 2.0) (Figure 3A). Our findings are in agreement with earlier studies that detected great variability in the gut archaeal population with age [37,38]. This suggests that constant exposure to various management factors and aging promote the colonization of the gut by other archaeome members and members of the gut microbiota [13].

Tulathromycin is a bacteriostatic macrolide with potential properties that are extensively utilized therapeutically in swine for the control or prevention of infectious disease [39]. Tulathromycin acts by inhibiting bacterial protein biosynthesis as well as inhibiting ribosomal peptidyl tRNA translation during the translocation process [40]. Sound scientific evidence shows that a TUL intervention has a negligible impact on microbiota establishment and their accompanying antimicrobial resistome [41], but its impact on archaeal populations has not been studied. In this study, there was no significant change detected in the archaeal genera that averaged more than 1% between the CONT and TUL groups. However, in-depth analysis at the genera level suggested that the treatment with TUL was associated with moderate changes in the archaeal profile in the feces of these young piglets. Compared with CONT piglets, the TUL-treated piglets on day 5 had a significantly lower abundance of *Archaeoglobus* (0.48% vs. 1.36% in CONT piglets, *p* = 0.041) and *Methanothermus* (0.1% vs. 0.69% in CONT piglets, *p* = 0.022) and a significantly higher abundance of *Nanoarchaeum* (0% vs. 0.014% in CONT piglets, *p* = 0.021). The TUL-treated piglets on day 20 showed a significantly higher abundance of *Halogeometricum* (0.289% vs. 0% in CONT piglets, *p* = 0.037) and *Acidilobus* (0.162% vs. 0.0001% in CONT piglets, *p* = 0.021) compared with the CONT group at the same time point. 

For further characterization of the differences in the fecal archaeal population between the two groups and to determine the indicator taxa in each group, the LEfSe algorithm was applied to measure the contribution of archaeal taxa in each group and identify the differences between the two groups. The LEfSe analysis revealed that the changes in the fecal archaeal structure caused by the perinatal TUL intervention were limited to a particular group of archaeal taxa (Figure 3B,C). Compared with the CONT group, two archaea were identified as indicator taxa in the TUL-treated piglets on days 5 and 20. On day 5, the TUL-treated piglets exhibited a high contribution of the *Nanoarchaeum* and *Nanoarchaeota* (Figure 3B). On day 20, *Halogeometricum* and *Acidilobus* were enriched in the TUL group (Figure 3C). Archaeal populations are characterized by a lack of peptidoglycan in their cell wall in addition to differences in DNA repair, genetic features, and biochemical and metabolic capabilities [42]. These unique phenotypic and genotypic characteristics make these microbes resistant to a wide spectrum of antimicrobial agents [43]. However, some archaeal populations are susceptible to antimicrobial agents, such as lipoglycopeptide, glycopeptides, and fosfomycin, that act on cell wall synthesis [44]. Therefore, defining how archaeal populations communicate with the host and respond to antimicrobial administration may help in addressing the role of the archaeome in both health and disease. Additionally, understanding the resistance and susceptibility of archaea to antimicrobials can be used to purpose protocols for the decontamination of complex microbiota for selective archaea isolation.

In conclusion, it seems that perinatal tulathromycin metaphylaxis had relatively minor effects on the developmental dynamics of the fecal archaeome composition and diversity in the neonatal piglets. While the results of this study could open a new avenue in understanding the impact of TUL administration on the early life developmental dynamics of the fecal archaeome in piglets, our analyses were performed on a relatively small number of animals per group. Therefore, further long-term studies across larger populations are recommended to determine the beneficial and/or the detrimental effects of early life antimicrobial prophylaxis on the fecal archaeome structure in pigs. The results of this study provide fundamental information about archaeome colonization and succession in neonatal piglets that can serve as a reference base for further investigation of the hitherto unexplored swine gut archaeome. Additionally, the current study provides unique opportunities for future in-depth taxonomic classification of the gastrointestinal archaeome and how it influences the host’s metabolism and immune system. Further additional work is required to determine at which stage of production the transition between the highly dynamic early archaeal population and the stable adult archaeal community occurs.

## Data Availability

To be publicly available, we deposited the whole-genome sequencing data in MG-RAST under the following accession numbers: mgm4779141.3 to mgm4779164.3.

## Supplementary Materials

The following are available online at https://www.mdpi.com/article/10.3390/ani11061825/s1, Figure S1: Rarefaction curves of sequences reads obtained from fecal samples from piglets in both control (CONT) and tulathromycin (TUL) groups at different sampling days 0, 5, and 20.
