# Supplementary material for: Metagenomic Analysis of the Fecal Archaeome in Suckling Piglets Following Perinatal Tulathromycin Metaphylaxis
Source: Animals (Basel). 2021 Jun 18;11(6):1825. doi: 10.3390/ani11061825 (PMC8235425; doi:10.3390/ani11061825)

Supplementary Material

Figure S1. Rarefaction curves of sequences reads obtained from fecal samples from piglets in both control (CONT) and tulathromycin (TUL) groups at different sampling days 0, 5, and 20.

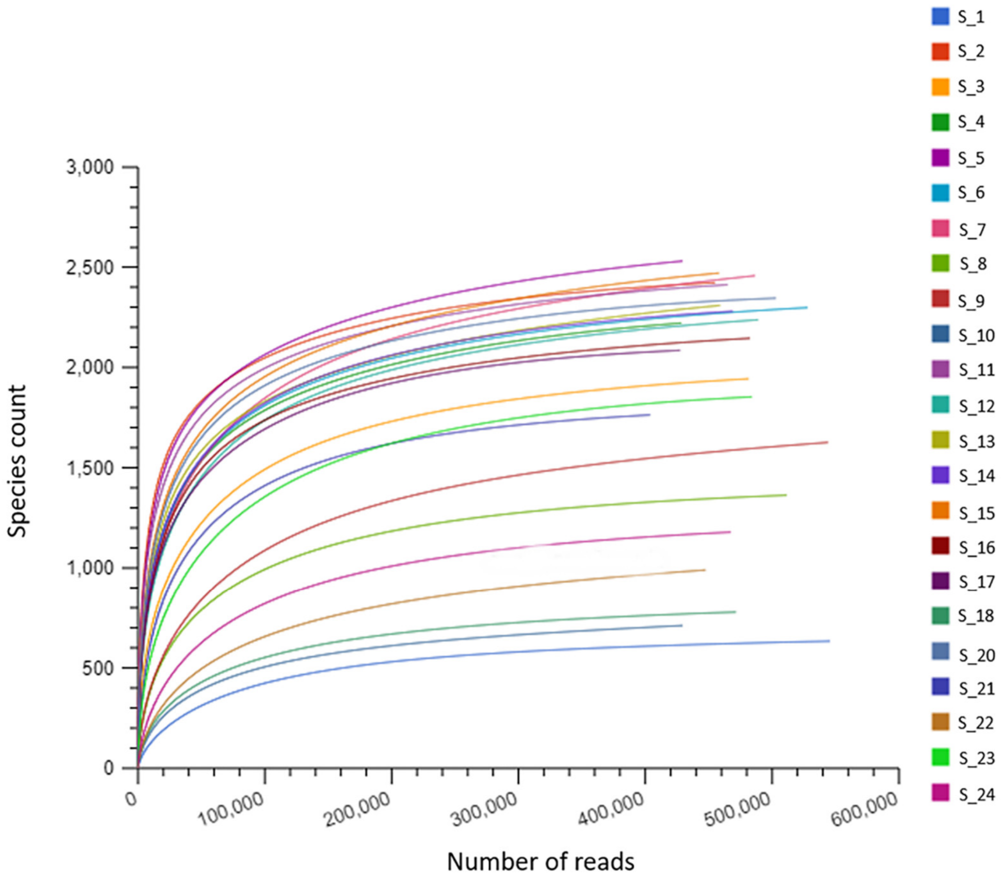

Supplement: Supplementary file 1 [file animals-11-01825-s001.zip › animals-1237493-supplementary.pdf]
